# Miscellaneous Facts and Remarks

**Published:** 1799-12

**Authors:** 


### *Miſcellaneous Facts and Remarks.*

Dr. FERRO, of Vienna, in conſequence of an Eſſay publiſhed in the 5th Number of the *Journal der Erfindungen*, relative to the treatment of the *nervous fever*, by Dr. BRANDIS, obſerves, that he has likewiſe found the *luke-warm bath* to be the ſafeſt and moſt effectual remedy againſt that fatal diſeaſe, which, at Vienna, every winter, deſtroys many perſons in the bloom and prime of life.—*Journ. der Erfind.* No. VI. p. 135.

---

Dr. L. FRANK, of Milan, coincides in opinion with Dr. Ferro, and has publiſhed, ſome time ago, in the Medical and Chirurgical Gazette of Salzburg, ſome excellent remarks *on the bracing virtues of the warm bath, and on Moſcati's mercurial calx.* The tonic powers attributed to the tepid bath by Dr. MARCARD, in his late and claſſical work, are particularly obvious in that ſingular diſeaſe called *Pellagra*, where great and general debility is the principal ſymptom.—Moſcati's oxyd of mercury conſiſts merely of calomel digeſted in an alkaline ley, and thus deprived of its acid, while it aſſumes a black colour. Dr. Frank prefers this to other mercurial preparations, becauſe it operates more gently, with equal ſafety, and is ſeldom attended with ptyaliſm. He alſo preſcribes it externally, in the form of mercurial ointment for friction.—*Ibid.* No. X. p. 133.

---

The medicinal virtues of certain vegetable ſubſtances (though we do not wiſh to increaſe the Materia Medica in quantity ſo much as in quality) certainly appear to deſerve every attention. Among this number are the leaves of the *Ilex Aquifolium*, LINN. The laſt treatiſe on this

ſubject

ſubject was publiſhed at Halle, in 1789, by A. D. BANDELOW, and entitled: *De foliorum Ilicis Aquifolii analyſi et virtute medica*, 8vo.—The author maintains, that the leaves of the holy-oak produce a tonic and reſolvent effect, that they promote ſecretions and evacuations, particularly by diaphoreſis. From one to two ounces of the leaves (it is not ſaid whether dry or freſh) are boiled in a quart of beer or water, and this decoction is gradually drank through the day. According to Mr. B. this remedy, in a ſhort time, cured obſtinate rheumatic and arthritic pains. He alſo recommends it for the cure of intermittents; and as we are frequently baffled in thoſe diſeaſes with our preſent ſtock, perhaps theſe leaves would deſerve a fair trial in this country, both on account of their cheapneſs, and the eaſy method of preparing them as a medicine.—*Ibid.* No. VI. p. 138.

---

Citizen BERLINGHERI has preſented to the Philomatic Society of Paris, three memoirs extracted from the works of Dr. CHIARENTI.—The firſt contains obſervations on the uſe of the gaſtric juice, in diſeaſes of the ſtomach. CHIARENTI ſucceſsfully repeated the experiments on digeſtion, made by SPALLANZANI and REAUMUR, and likewiſe attempted ſome new ones: he treated ſeveral diſeaſes of the ſtomach with ſucceſs, by adminiſtering the gaſtric juice of a variety of animals in different doſes. The ſecond memoir relates to opium, and its action on the animal œconomy.—CHIARENTI was of opinion, in conſequence of repeated experiments, that opium does not operate like common emetics; and that it continues inactive, till, having combined with the gaſtric juice in the ſtomach, it is digeſted and paſſed into circulation. Conſiſtently with this conjecture, he formed a mixture of opium, of gaſtric juice, and pomatum, which he applied by friction, on the ſkin of man and animals; and in this manner he aſcertained the effects of opium taken internally. Thus, opium may be adminiſtered in the moſt critical diſeaſes of the ſtomach, and even to infants of the moſt tender age. CHIARENTI alſo applied it with ſucceſs, as a local remedy, in acute diſeaſes; ſuch as the *gout*, the *odontalgia*, and even in diſeaſes of the breaſt. The third memoir is an attempt to prove that the gaſtric juice is deſtined by Nature to promote the abſorption of many ſubſtances. He mixed the gaſtric juice with ſquills, rhubarb, and bark; and the frictions with theſe mixtures produced effects ſimilar to thoſe reſulting from theſe ſubſtances, when taken internally.

Several Italian phyſicians, in repeating theſe experiments, have obſerved ferved analogous effects: fome of them attempted to fubftitute faliva, inftead of the gaftric juice, which, according to their affertion, anfwered the fame purpofe: hence they conclude, that all the animalifed liquids might afford the neceffary modification to medical fubftances, to introduce them by the cutaneous veffels.

Citizens ALIBERT and DUMERIL have repeated thefe experiments, at the requeft of the Philomatic Society. They adminiftered feveral purgatives by friction, and the bark thus applied has fucceeded in the treatment of obftinate quartan fevers; the fquill cured a child, the whole of whofe body was œdematous. They are now continuing thefe experiments, and hope to be able to announce, that the gaftric juice has no influence whatever on the effects of remedies, and that any other vehicle may be fubftituted.—*Rapport General des Travaux de la Societé Philomatique de Paris*, Vol. I. p. 132.

---

Citizen MARSILLAC read a memoir to the Society, refpecting a man, who, with a view to inoculate his two children, fpread fome fcabs of the variolous matter between flices of bread and butter; the children ate it, and gave a piece to a dog; they had a very mild fmall-pox, and the dog, at the end of the 4th day, had a complete variolous eruption: on the 9th the puftules were at their height, foon became dry, and fell off in the ufual manner. An Englifh Author has afferted, that he obferved the variolous epidemic among a flock of fheep; but perhaps this author has confounded the fcab with the fmall-pox, as there is a great analogy between thefe two difeafes.—*Ibid.* p. 136.

---

Citizen CHAUSSIER addreffed fome obfervations to the Society, on a fpecific againft the hydrophobia, fold at Paris and Lyons, under the name of the *Ormfkirk Remedy.* He obferves, that this pretended fpecific has long been employed in England, under the infpection of feveral of the moft celebrated phyficians, and always without fuccefs. He adds, that its compofition is known: It is prepared of an abforbent earth, the recipe of which is contained in the "Refearches of Citizen Andry, on Hydrophobia." Chauffier is of opinion, that the only means of preventing the confequences of this difeafe is to check the abforption of the virus, by cauterifing the bitten part, and producing an abundant fuppuration.—*Ibid.* p. 135.

---

CITIZEN BELLOT tranfmitted fome obfervations on hydrophobia; in in which he conſiders ſoap as the beſt preſervative and curative ſubſtance for the bite of mad animals. He cited ſeveral inſtances, in which the application of a lixivium of ſoap and water proved ſucceſsful.—*Ibid.* p. 130.

---

The ſame member alſo ſent ſeveral memoirs on various diſeaſes of patients admitted into the hoſpitals of Laon and Senlis, in the ſecond year of the Republic, particularly a caſe of *aſcites,* the ſubject of which had ſuddenly ſwelled to a dreadful ſize; but the ſwelling diſappeared in fifteen days, after an application of two large bliſters to the thighs, and the adminiſtration of an emetic, compoſed of two grains of tartarized antimony, and half an ounce of the ſulphat of ſoda in a little milk; with this treatment, he conjoined the tonic pills of Bacher.—*Ibid.*

---

Citizens LEVEILLE and LARREY made reports on the uſe of the muriat of barytes. The former employed it in the treatment of an *oſteo-ſarcoma* in the lower part of the leg, on which this ſalt, taken internally by the patient, produced no good effect. The latter aſſerted, that this ſubſtance was inefficacious in the treatment of ſcrofulous tumours. A patient, after having taken it during forty days, experienced diſtreſſing ſymptoms in the ſtomach, accompanied with an obſtinate diarrhœa, and a fever which continued while that remedy was adminiſtered, leaving him in a ſtate of great debility.—*Ibid.*

---

Citizen ALIBERT read a memoir on odours, and their effects as medicines. He mentioned ſeveral facts to which he was a witneſs, proving the multiplied relations between odours and the different morbific ſtates of the human body, particularly their influence on hyſterical and conſumptive diſeaſes. He is of opinion that this object is worthy of the reſearches of phyſicians and philoſophers, and ſolicited the Society to take notice of the corroborating accounts of others, and determine accordingly.—*Ibid.* p. 131.

---

Citizen BENON read a memoir on the advantages of treating venereal diſeaſes by corroſive ſublimate applied externally, and by injections. He was induced to judge of the good effects of this remedy, in conſequence of the ſucceſsful treatment of a delicate woman, who had a confirmed venereal diſeaſe.—*Ibid.* p. 136.

MEDICAL

